# Private Data Incrementalization: Data-Centric Model Development for Clinical Liver Segmentation

**DOI:** 10.3390/bioengineering12050530

**Published:** 2025-05-15

**Authors:** Stephanie Batista, Miguel Couceiro, Ricardo Filipe, Paulo Rachinhas, Jorge Isidoro, Inês Domingues

**Affiliations:** 1 Polytechnic University of Coimbra, Rua da Misericórdia, Lagar dos Cortiços, S. Martinho do Bispo, 3045-093 Coimbra, Portugalcouceiro@isec.pt (M.C.); 2 Institute of Applied Research (i2A), 3045-093 Coimbra, Portugal; 3 Laboratory for High Performance Computing (LaCED), 3030-199 Coimbra, Portugal; 4 Laboratory of Instrumentation and Experimental Particle Physics (LIP-Coimbra), Rua Larga da Universidade de Coimbra, 3004-516 Coimbra, Portugal; 5 Altice Labs, S.A, 3810-106 Aveiro, Portugal; ricardo.angelo.filipe@gmail.com; 6 Coimbra Hospital and University Center, 3004-561 Coimbra, Portugal; rac@chuc.min-saude.pt (P.R.); jisidoro@ulscoimbra.min-saude.pt (J.I.); 7 Medical Physics, Radiobiology and Radiological Protection Group, Research Centre of the Portuguese Institute of Oncology of Porto (CI-IPOP), 4200-072 Porto, Portugal

**Keywords:** private data incrementalization, artificial neural networks, U-Net, ResUNet++, FCN, BerDiff, computed tomography, automatic liver segmentation

## Abstract

Machine Learning models, more specifically Artificial Neural Networks, are transforming medical imaging by enabling precise liver segmentation, a crucial task for diagnosing and treating liver diseases. However, these models often face challenges in adapting to diverse clinical data sources as differences in dataset volume, resolution, and origin impact generalization and performance. This study introduces a *Private Data Incrementalization*, a data-centric approach to enhance the adaptability of Artificial Neural Networks by progressively exposing them to varied clinical data. As the target of this study is not to propose a new image segmentation model, the existing medical imaging segmentation models—including U-Net, ResUNet++, Fully Convolutional Network, and a modified algorithm based on the Conditional Bernoulli Diffusion Model—are used. The study evaluates these four models using a curated private dataset of computed tomography scans from Coimbra University Hospital, supplemented by two public datasets, 3D-IRCADb01 and CHAOS. The *Private Data Incrementalization* method systematically increases the volume and diversity of training data, simulating real-world conditions where models must handle varied imaging contexts. Pre-processing and post-processing stages, incremental training, and performance evaluations reveal that structured exposure to diverse datasets improves segmentation performance, with ResUNet++ achieving the highest accuracy (0.9972) and Dice Similarity Coefficient (0.9449), and the best Average Symmetric Surface Distance (0.0053 mm), demonstrating the importance of dataset diversity and volume for segmentation models’ robustness and generalization. *Private Data Incrementalization* thus offers a scalable strategy for building resilient segmentation models, ultimately benefiting clinical workflows, patient care, and healthcare resource management by addressing the variability inherent in clinical imaging data.

## 1. Introduction

The integration of artificial intelligence (AI), particularly Machine Learning (ML) algorithms, in healthcare has the potential to revolutionize diagnostic and treatment processes, particularly in medical image segmentation. One of the main challenges in the medical field is the accurate and efficient segmentation of organs, such as the liver, in imaging modalities like computed tomography (CT) [1]. This task is essential for diagnosing, planning, and monitoring treatments, including those in radiotherapy and nuclear medicine, where precise organ boundaries are necessary for treatment planning. However, the current commercial solutions for liver segmentation are often expensive, and free alternatives can lack reliable performance and replicability.

In response to these challenges, the development of cost-effective and efficient solutions using segmentation methods, more specifically Artificial Neural Networks (ANNs), is crucial [2]. Nevertheless, the success of ANNs in medical image segmentation is heavily influenced by several factors, including the volume, resolution, and source of the data used for training. Many models show poor generalization capability when applied to clinical data from sources different from the ones used for the initial training, which compromises their robustness in real-world settings [3,4]. Second, there is a strong dependency on large volumes of high-quality annotated data, which is not always feasible due to privacy concerns, equipment heterogeneity, and differences in clinical protocols [5,6]. Finally, the current methods tend to treat datasets as fixed homogeneous blocks, overlooking scenarios where clinical data become available incrementally—a setting that better reflects the reality of hospital environments.

Among the most prominent strategies to mitigate data scarcity and privacy concerns are Federated Learning (FL) and the generation of synthetic data. FL enables decentralized training across multiple institutions without requiring data centralization, thus preserving patient confidentiality [7,8]. However, FL introduces non-trivial technical and operational complexities, such as handling non-IID (non-independent and identically distributed) data, ensuring synchronization and communication between nodes, and requiring homogeneous infrastructure across institutions—conditions that are often unfeasible in diverse clinical environments.

The generation of synthetic data, through Generative Adversarial Networks (GANs) [9] or diffusion models, can increase the volume of training data. Nevertheless, these methods may introduce artifacts or generate unrealistic samples that fail to capture the full clinical variability required for high-stakes applications such as organ segmentation.

The approach proposed in this study—*Private Data Incrementalization* (*PDI*)—constitutes a pragmatic and data-centric alternative. Rather than relying on distributed infrastructures or synthetic data, *PDI* assumes that real institution-specific data become gradually available over time. This paradigm enables the progressive refinement of models, offering improved adaptability within realistic clinical constraints. Importantly, it facilitates continuous model enhancement without the ethical and operational burdens typically associated with federated or generative approaches.

This paper presents the concept of *Private Data Incrementalization* (*PDI*), a data-centric approach that compares the performance of ML models when introducing diverse real-world clinical data to model training. The objective is to evaluate how such incremental exposure enhances the adaptability and generalization capabilities of segmentation models. The methods undertaken to achieve that objective include the initial training of the model with publicly available data and re-training with increasingly specific data, mimicking a real-world scenario where targeted data become available through time. Since this study does not aim to propose a new image segmentation model, the experiments are conducted using existing medical imaging segmentation models.

In this way, the contributions of the present work can be summarized as follows:

- The definition of the concept of *Private Data Incrementalization* (*PDI*);
- The exemplification of the *PDI* concept in a liver segmentation scenario on CT scans (keeping in mind that this is only a possible use case, while the *PDI* idea is much more generic);
- Extensive experiments using several ANN models, including U-Net, ResUNet++, Fully Convolutional Network (FCN), and a modified algorithm based on the Conditional Bernoulli Diffusion Model (BerDiff);
- Evaluation with a comprehensive set of metrics to characterize the several dimensions of the problem.

This paper is divided into six additional sections: Section 2 contextualizes the state of the art in liver segmentation; Section 3 introduces the concept of *PDI*; Section 4 details the methodology adopted for this study; Section 5 presents the quantitative and qualitative results obtained by the different experiments; Section 6 discusses the results and relevant findings; and Section 7 summarizes the conclusions, strengths, limitations, and the directions for future work.

## 2. State of the Art

This review focuses on the four architectures used in the experimental part of the work: U-Net, ResUNet++, FCN, and BerDiff. For a broader literature review and historic perspective on the topic of liver segmentation, we point to [1]. The search was conducted using *Publish or Perish* (version 8.16.4790.9060). Search term “liver segmentation computed tomography MODEL”, where MODEL is one of the four architectures, was retrieved from the keywords of the papers. The data were collected from Google Scholar on 13 November 2024, excluding state-of-the-art reviews and citations to focus solely on original research articles. The search parameters included filtering results by the specified keywords and limiting the publication years to 2024. Only results with over 10 citations are included here. The literature review was then expanded to include more recent advancements in computer vision, in particular the integration of transformer-based approaches, combination of convolutional and transformer architectures, and foundation models.

The patent presented by Paik et al. (2024) [10] proposes the application of advanced AI and ML algorithms for complex medical image data analysis based on techniques including Convolutional Neuronal Networks (CNNs), more specifically Fully Convolutional Networks (FCNs) using cross-entropy loss with an Adam optimizer for the segmentation of cervical, thoracic, and lumbar spine imaging studies such as MRI and CT scans, emphasizing the performance in tasks like segmentation and diagnosis. While promising, the document highlights the need for adaptive algorithms that handle variability in imaging data, although it may not fully detail the extent of variability these adaptive algorithms can manage or the specific mechanisms for achieving such adaptability in diverse clinical scenarios.

The TransUNet model is employed by Chen et al. (2024) [11], leveraging transformers designed for sequence-to-sequence predictions for long-range dependency modeling within the U-Net framework, encapsulating transformers’ self-attention regarding two key modules, the first one consisting of a transformer encoder tokenizing image patches from a CNN feature map, facilitating global context extraction, and the second one including a transformer decoder refining candidate regions through cross-attention between proposals and U-Net features, resulting in three configurations: encoder-only, decoder-only, and encoder+decoder, achieving average DSC values of 0.8811, 0.8763, and 0.8839, respectively, for multi-organ segmentation, encouraging the adoption of transformers in medical applications, offering both 2D and 3D implementations. However, the computational overload associated with transformer mechanisms, especially for high-resolution 3D medical images, can be substantial, and strategies for optimizing these models for real-time clinical deployment may require further exploration.

The framework proposed by Sun et al. (2024) [12] introduces the application of the DA-TransUNet segmentation model, integrating dual attention mechanisms with transformers into the U-Net architecture. This model focuses on spatial and channel dual attention blocks (DA blocks) within skip connections to enhance feature extraction and filter irrelevant information. The DA-TransUNet achieves superior performance across five datasets compared to the other state-of-the-art methods, obtaining 0.7980 of DSC, in comparison to a DSC of 0.7584 by TransUNet. The authors share some limitations and challenges of the study, including the increased complexity due to the introduction of DA blocks.

A 3D large-kernel (LK) attention module is introduced by Li et al. (2024) [13]. The proposed approach consists of the combination of self-attention with convolution, where the LK module captures long-range dependencies, local contextual information, and channel adaptation, integrating into a CNN, more specifically the U-Net architecture, to reduce the disadvantage of high computational cost. The model achieves state-of-the-art performance on public datasets, considering the focus on optimizing computational efficiency for real-time clinical applications relevant for future improvements, with, taking into account the results presented, mean averaged DSC values of 0.8404 and 0.8481 for the base and mid methods presented for multi-organ segmentation. However, the authors mention some challenges for the study, including the need for better sampling or training strategies for large medical images to improve the resolution of the segmentation results, and the low quality of some images, which reduces segmentation performance, indicating a sensitivity to data quality that might limit broader applicability without extensive pre-processing or data curation.

The authors Ma et al. (2024) [14] present the U-Mamba approach with long-range dependency modeling in biomedical image segmentation by combining CNNs with State Space Models (SSMs). This hybrid architecture uses Mamba blocks to balance localized feature extraction with global context understanding applied to 3D abdominal organ segmentation in CT and Magnetic Resonance Imaging (MRI) images, instrument segmentation in endoscopy images, and cell segmentation in microscopy images, using “U-Mamba_Bot” and “U-Mamba_Enc” to differentiate the two network variants employing the U-Mamba block in the bottleneck and all the encoder blocks, respectively. The U-Mamba approaches surpassed both CNN and transformer-based segmentation networks, achieving average DSCs of 0.8683 (U-Mamba_Bot) and 0.8501 (U-Mamba_Enc) on the abdomen CT and MR datasets, respectively, concluding that the U-Mamba model achieves positive results across diverse datasets, providing an efficient alternative to transformer-heavy architectures, although it still needs to be improved to address scalability for higher-resolution medical images, and its performance on datasets with large domain shifts remains to be fully characterized.

Banerjee et al. (2024) [15] focus on an analysis of the integration of physical laws into computer vision models to enhance interpretability, robustness, and data efficiency. By embedding physics-informed priors into various stages of the vision pipeline, the approach improves alignment with real-world phenomena, presenting an external study that adopts a physics-informed Machine Learning (PIML) segmentation approach in medical scans that raises the DSC values for gray matter from 0.904 to 0.910 and for white matter from 0.943 to 0.948. While promising for tasks like image segmentation, the current challenges include the standardization of benchmarks and the incorporation of physics into tasks such as human motion tracking, and the generalizability of specific physics-informed priors to a wide range of medical imaging modalities and conditions can be limited.

The approach of Jiao et al. (2024) [16] involves the building of a Universal Ultrasound Foundation Model (USFM) to address label efficiency and task adaptability in ultrasound image analysis. By pre-training on a dataset of multi-organ images and using a spatial-frequency dual masked image modeling method, the model extracts features from low-quality US images. The authors conclude that the USFM achieved high performance with minimal annotations, making it a strong candidate for multi-organ US segmentation, by obtaining DSCs of 0.8590 for the thyroid segmentation and 0.8430 for the breast segmentation. The model’s scalability and efficiency make it a promising solution, although challenges in insufficient public datasets, images with low quality, ineffective features, and model generalization remain. The nature of such foundation models for highly specialized tasks like ultrasound, which suffers from operator-dependent variability, still requires extensive validation across diverse clinical settings and equipment.

Liu et al. (2024) [17] describe an analysis of the application of the probabilistic generative model presented as the denoising diffusion probabilistic model (DDPM) in comparison with other methods for medical image segmentation, showing how the DDPM performed in denoising and capturing complex data distributions, addressing challenges in imaging variability and noise. The paper underscores the DDPM’s potential in advancing reliable medical imaging techniques while calling for more research on scalability and real-world implementation, achieving a 0.920f DSC, which indicates positive results. However, other methods studied within the context of the paper also present good performance, including BerDiff, Collectively Intelligent Medical Diffusion (CIMD), Adaptive Mask Segmentation Attack (ASMA), and DermoSegDiff. While DDPMs show promise, their iterative denoising process can be computationally intensive, and their application to complex 3D segmentation tasks often requires architectural adaptations or simplifications, as noted with BerDiff in our study.

Further expanding on architectural advancements, the evolution of transformer-based models like detection transformer (DETR) [18] and its subsequent refinements such as Deformable DETR [19] have impacted object detection. While primarily focused on detection, the principles of using transformer encoders and decoders for global context modeling and feature refinement are increasingly influential in segmentation tasks. For instance, approaches like DINO (DETR with Improved DeNoising) [20] further improve DETR by addressing training stability and performance. These advancements highlight a trend towards more integrated and powerful attention-based architectures, although their direct application to volumetric medical image segmentation often requires modifications to handle 3D data efficiently and manage computational costs.

Specifically for medical image segmentation, hybrid models combining the strengths of CNNs and transformers are gaining traction. For example, Ref. [21] proposes MAFT-Net (multi-scale attention frequency-enhanced transformer network), which integrates multi-scale features from CNNs with a frequency-enhanced attention transformer, allowing the model to capture both local texture details and global contextual information, with frequency domain processing helping to enhance the relevant features. Such hybrid approaches aim to overcome the limitations of using either CNNs or transformers in isolation, but they can also introduce increased model complexity and a larger number of hyperparameters, requiring careful tuning.

Techniques such as transformer integration in DA-TransUNet and TransUNet allow for the modeling of long-range dependencies, whereas U-Mamba combines CNNs with state-space models for efficient global context understanding. Generative approaches, such as BerDiff’s application of DDPMs, further increase robustness against image variability and noise. These innovations achieve high DSCs, demonstrating their effectiveness on diverse datasets and imaging modalities. Nevertheless, many models face difficulties, including increased computational demand for attention mechanisms, insufficient public datasets for robust training, and limited generalization across imaging modalities and clinical applications. Scalability for high-resolution imaging and in the absence of standardized benchmarks also makes broader adoption difficult.

The broader landscape of medical AI is also shifting towards foundation models, as reviewed by [22,23]. These models, pre-trained on vast and diverse datasets, aim to provide a base for a wide range of downstream tasks, including segmentation, through fine-tuning. While the development of true foundation models for medical imaging is still in its early stages due to challenges in data access, privacy, and heterogeneity, their potential to improve generalization and reduce the need for extensive task-specific training is high. However, adapting these large-scale models to specific clinical applications, like precise liver segmentation with limited institutional data, remains a key challenge, underscoring the need for effective fine-tuning and incremental learning strategies. Addressing these issues would require optimizing lightweight architectures, increasing the quality of datasets and developing unified evaluation frameworks [24], and devising robust strategies for model adaptation to specific clinical environments. The present paper makes a contribution to addressing the problem of limited diversity of datasets and model adaptation through Private Data Incrementalization, as described next.

## 3. Private Data Incrementalization

*PDI* consists of a systematic approach intended to improve the adaptability of segmentation models by incrementally exposing them to more specific and targeted data. This approach acknowledges the variability inherent in clinical imaging data and seeks to closely reproduce real-world conditions in which hospitals have equipment that differs in advancement, influencing quality and the resulting exams, by combining private and public data during model training, having different sources, and also different strategies for creating ground truth, which represents one of the main challenges of a real-world scenario where there is subjectivity when it comes to segmentation acceptability. In this way, different experts can have different degrees of agreement with a segmentation.

This approach is driven by the difficulties faced by segmentation models when applied to datasets with different image characteristics, such as resolution, contrast, and patient demographics. These variations can lead to a decrease in model performance, especially in clinical scenarios where models are faced with unseen data. *PDI* potentially provides a framework to mitigate this by ensuring structured exposure to data, allowing for better adaptability.

**Figure 1:**
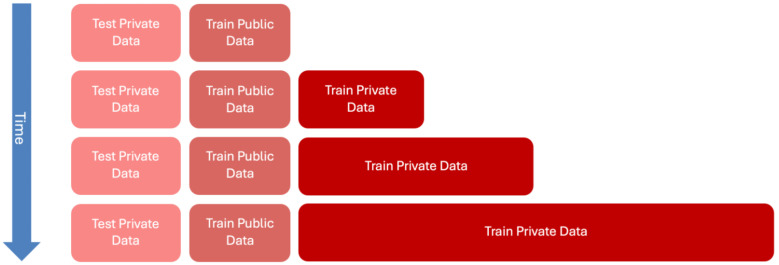
Structure of the *Private Data Incrementalization* (*PDI*) process. Across the layers, the structure remains consistent, but the proportion of private data in the training set increases incrementally to reflect the progressive availability of clinical data in real-world settings.

The techniques consist of incrementally increasing the volume of the training data through a gradual integration of more targeted data, evaluating how the data sources and their volume can influence the performance of the models. This is illustrated in Figure 1. In this figure, although the components remain the same across the different layers, their proportions vary, with private data becoming increasingly prevalent in the training process.

This methodology emulates a real-world scenario where specific data are initially scarce, requiring the use of available generalized data to train the models. Over time, as more specific and targeted data become progressively available, the models can be re-trained to improve their performance. The aim is to strike a balance by identifying the optimal point at which additional data or further training no longer enhances the model’s accuracy or utility.

## 4. Methodology

The methodology included the study of the clinical and AI workflows (Section 4.1), design of the experiential setup (Section 4.2), data preparation (Section 4.3), definition of the segmentation pipeline (Section 4.4), experimental specification (Section 4.5), model configuration (Section 4.6), and performance evaluation (Section 4.7), focusing on models under varying data volumes and sources. This structured approach ensures that the models are exposed to diverse clinical scenarios with the objective of developing robust segmentation models that can generalize to new unseen data.

### 4.1. Overview

**Figure 2:**
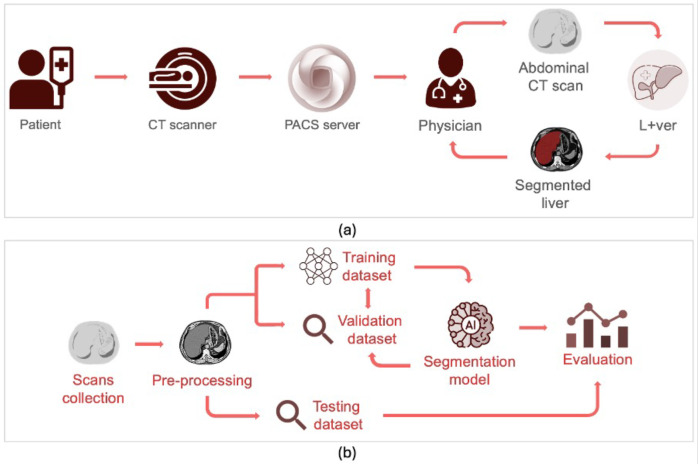
Methodology of the proposed study: (**a**) clinical workflow; (**b**) AI workflow.

The objectives of the project led to the creation of the workflow diagrams shown in Figure 2, which illustrate the process from a clinical perspective (Section 4.1.1) and from a technical perspective (Section 4.1.2).

#### 4.1.1. Clinical Workflow

The clinical workflow is composed of the following elements:

- **Patient:** the process starts with a patient who needs an abdominal CT scan.
- **CT scanner:** the patient undergoes a CT scan to obtain detailed images of the abdominal area.
- **Picture Archiving and Communication System (PACS):** the CT scan is stored on the PACS server. PACS is a system used in medical imaging to store, retrieve, and share images efficiently, making it easier for healthcare professionals to collaborate and improve patient care.
- **Physician:** a physician accesses the CT scan images from the PACS server.
- **Upload CT scan:** the physician uploads the abdominal CT scan for liver identification and segmentation.
- **L+ver (liver segmentation app):** the presented solution automatically segments the liver from CT images for further analysis or treatment planning.
- **Evaluation and decision:** the segmented liver images are evaluated by the physician to make a treatment decision.

#### 4.1.2. Artificial Intelligence Workflow

The focus of this study is on the AI workflow, which is the main component of the L+ver app. Although the app also includes additional features to visualize medical images, these are not the main subject of this research. The AI workflow is composed of the following steps:

- **Scan collection:** the workflow begins with the collection of abdominal CT scans.
- **Pre-processing:** the collected scans are pre-processed to ensure they are suitable for training an AI model.
- **Training and validation dataset:** the pre-processed scans are divided into a training and a validation dataset. The training dataset is used for the model to learn patterns, relationships, and features. The validation dataset is used to fine-tune the model, helping to prevent overfitting and ensuring that it generalizes well to unseen data.
- **Segmentation model:** the AI segmentation model is trained using the augmented training dataset.
- **Testing dataset:** a separate testing dataset is used to validate the effectiveness of the model.
- **Final evaluation:** the performance of the AI model is thoroughly evaluated to ensure it meets the required standards for clinical use.

An additional step implemented was post-processing, which aimed to optimize the outcome (see Section 4.4.3).

### 4.2. Experimental Setup

In the experimental setup, four architectures were chosen: U-Net, ResUNet++, FCN, and BerDiff. Each model underwent five experiments using different dataset configurations to test its adaptability and performance. These experiments evaluated each model’s accuracy, precision, and Dice Similarity Coefficient (DSC) across configurations, particularly focusing on metrics relevant to clinical segmentation tasks. For the reader interested in learning more about the metrics, including their equations, we point to [24,25]. An early stopping mechanism based on validation loss was applied to prevent overfitting, ensuring that models maintained generalization capability.

All experiments were performed on GPU resources to handle the computational demands of the model architectures and dataset sizes, and training times were tracked to assess the computational efficiency of each model under varying data volumes. All experiments were implemented using a NVIDIA^®^ T4 Tensor Core GPU and a NVIDIA^®^ A100 GPU, with Kaggle and Google Colab Pro+ serving as the programming environments, respectively.

### 4.3. Data Preparation

To carry out the project, three datasets were used, whose comparison can be found in Table 1, highlighting the extensive volume of the datasets and their main characteristics.

**Figure 3:**
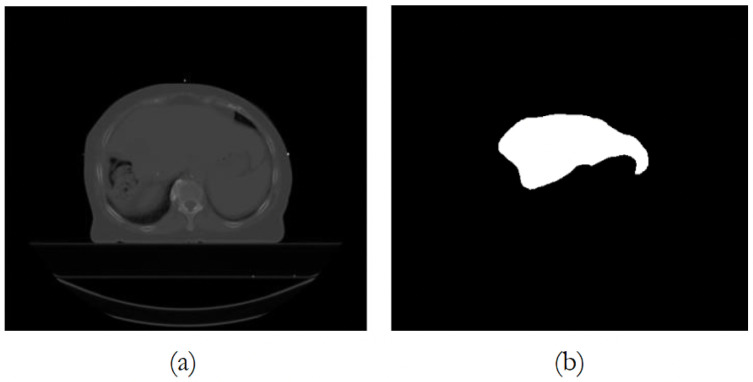
Example of a CT scan from the private dataset: (**a**) CT scan image/slice; (**b**) liver segmentation ground truth mask.

The private dataset was provided by the *Hospitais Universitários de Coimbra* (HUC) Radiotherapy Department. All scans are anonymized, and the slices/images have been reviewed to preclude the presence of personal information. Table 2 presents descriptive statistics for the ages of the participants, and an illustrative image is given in Figure 3. For more details on the private dataset, we refer to [28].

The 3D-IRCADb01 public dataset is composed of the 3D CT-scans of 20 patients with hepatic tumors in 75% of cases. CHAOS challenge aimed at the segmentation of abdominal organs (liver, kidneys, and spleen) from CT and MRI scans. CHAOS was held in the IEEE International Symposium on Biomedical Imaging (ISBI) on 11 April 2019, Venice, Italy. For this challenge, a public dataset was presented [27]. This dataset contains 80 medical imaging scans, but only 40 of them are CT scans from healthy abdomen organs without any pathological abnormalities; the remainder consisted of MRI scans. From the 40 CT scans, only 20 were intended by the challenge to be used for training and the remaining 20 for testing, with no ground truth for the latter. Given the methodology defined for implementing this project, it was decided to discard the 20 test CT scans.

In this paper, each phase of incrementalization introduces new data from the private dataset according to the following structure:

- In the initial phase, models are trained exclusively on private data to establish a baseline performance.
- In a subsequent phase, only public datasets are used to simulate various imaging conditions.
- The final phases merge private and public datasets, providing a comprehensive and varied training environment.

**Figure 4:**
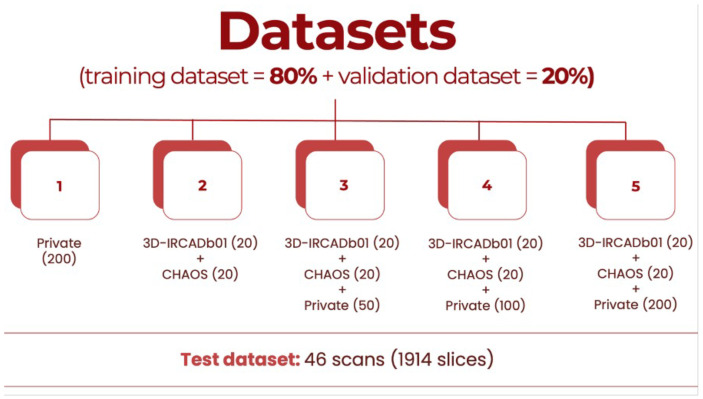
Datasets’ distribution based on number of scans.

Figure 4 presents the distribution and structure of each dataset used to train, validate, and test the models. Slices with no liver were not used.

### 4.4. Segmentation Pipeline

**Figure 5:**
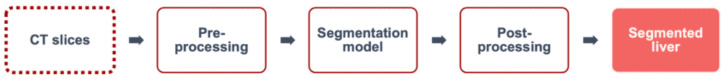
Segmentation flowchart.

The overall segmentation pipeline is shown in Figure 5 and includes data pre-processing (Section 4.4.1), segmentation models (Section 4.4.2), and post-processing (Section 4.4.3).

#### 4.4.1. Pre-Processing

**Figure 6:**
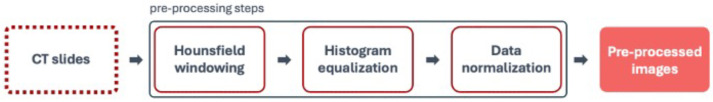
Pre-processing flowchart.

Pre-processing in medical imaging is a crucial step to prepare images for further analysis. This phase involved various techniques to enhance the quality and usability of the images. Figure 6 represents the steps for the pre-processing stage.

Hounsfield windowing, also known as window-levelling or contrast stretching, is a technique used in CT imaging to enhance the visibility of specific tissues or structures. By focusing on a particular range of HU values, clinicians can better visualize ROIs [29,30]. Specifically, using a window range of −100 to 400 HU was effective for visualizing soft tissues and some bony structures. This interval was proposed by Sabir et. al. [31] for their liver segmentation proposal, with the aim of optimizing the correct visualization not only of the liver but also of other structures. This range included most organs, muscles, fat, and blood, enhanced lung details at the lower end (−100 HU), and captured less dense bones at the upper end (400 HU).

Histogram equalization is a technique to improve image contrast by spreading out the most frequent intensity values. This process makes hidden features more visible, enhancing the quality of images [32,33]. This technique proved particularly useful for images with poor contrast, often dominated by bright or dark regions, and, in medical imaging, it helped to make important structures within tissues or organs more visible.

Data normalization involves scaling the numerical data features to a uniform range. This process ensures that all features have similar scales, facilitating the ability of the segmentation algorithms to process the data effectively and produce accurate segmentation results. By transforming each feature so they fall within a common range, such as 0 to 1, normalization prevents any single feature from dominating the learning process due to its larger scale. This uniformity is crucial for the ANNs to treat all features equally, leading to improved performance and more precise liver segmentation in the CT scans.

#### 4.4.2. Segmentation Models

For model selection, four architectures were chosen: U-Net, ResUNet++, FCN, and BerDiff. These models were selected based on their established performance in medical image segmentation and their architectural diversity, offering a broad range of techniques for addressing segmentation challenges.

U-Net, with a total of 1,940,817 parameters, is structured in a U-shape to efficiently use computing resources while balancing the depth of the network. The architecture consists of a contracting path, also known as the encoder, to capture the context of the image and a symmetric expanding path, known as the decoder, for precise localization. The encoder path includes convolutional layers with ReLU activation and max-pooling layers to downsample the input image, capturing the context in lower resolutions. The decoder path, on the other hand, consists of up-convolution layers to upsample the features, restoring the spatial resolution and enabling the delineation of the liver boundaries [34].

ResUNet++, with a total of 4,062,673 parameters, is a CNN whose architecture builds on the U-Net and ResUNet models, incorporating residual blocks, squeeze and excitation blocks, and an Atrous Spatial Pyramid Pooling (ASPP) bridge, which collectively enhance the ability of the model to capture and process detailed spatial and contextual information, and attention mechanisms to enhance the accuracy of pixel-wise segmentation [35]. The architecture of ResUNet++ consists of three main parts: encoding, bridge, and decoding. In the encoding phase, the model processes the input through a series of Conv2D layers (3×3 filters), batch normalization, and ReLU activations. Residual connections and squeeze-and-excite blocks enhance feature representation and channel-wise feature recalibration. The bridge section employs an ASPP layer to capture multi-scale contextual information. The decoding phase involves a sequence of Conv2D layers, batch normalization, ReLU activations, and attention mechanisms. Upsampling layers are used to restore the spatial resolution, and concatenation merges features from corresponding encoding stages. The final output is obtained through a sigmoid activation, indicating the segmentation map.

The chosen FCN architecture, with a total of 134,264,538 parameters, is based on a VGG-like encoder–decoder structure. The encoder consists of 5 convolutional blocks, each followed by a MaxPooling2D layer, progressively reducing the spatial dimensions of the input while increasing the depth of feature maps. The final layers of the encoder are fully convolutional, with two Conv2D layers using large filters (4096 units) to capture more abstract features. In the decoder, upsampling is performed through Conv2DTranspose layers, gradually restoring the original image size. Skip connections from the encoder’s corresponding layers are added at each upsampling stage to retain fine spatial details. This architecture allows FCN to make predictions for every pixel in the input image, transforming the network from a classifier to a semantic segmentation model [36].

BerDiff is a Conditional Bernoulli Diffusion Model developed for medical image segmentation, addressing challenges such as inherent ambiguity and high uncertainty in medical images. Unlike traditional diffusion models that use Gaussian noise, BerDiff employs Bernoulli noise as the diffusion kernel, enhancing the ability of the model to produce accurate binary segmentation masks. The architecture of BerDiff includes a forward diffusion process, where Bernoulli noise is progressively added, and a reverse process that iteratively refines the segmentation masks. This approach allows BerDiff to generate multiple diverse segmentation masks from the same input image, providing valuable references for radioncologists. Additionally, BerDiff incorporates efficient sampling techniques, inspired by denoising diffusion probabilistic models and denoising diffusion implicit models, to speed up the segmentation process [37]. When training the BerDiff original model, with a total of 1,869,489 parameters, a RuntimeError: CUDA out of memory was obtained. As a result, it was necessary to adapt and simplify the BerDiff model to a total of 577,667 parameters. The strategies applied to reduce the memory usage and complexity of the BerDiff model included the following:

- Reduction in the number of down/upsampling layers from 3 to 2.
- Reduction in the number of channels from 64 to 32.
- Use of fewer layers.
- Implementation of gradient check-pointing, which trades computation for memory by recomputing intermediate activations during the backward pass.

#### 4.4.3. Post-Processing

**Figure 7:**
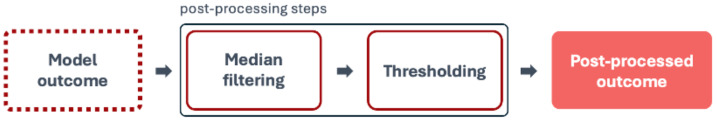
Post-processing flowchart.

In medical imaging, post-processing techniques are helpful procedures for analyzing model-generated images in order to enhance accuracy and overcome limitations. These techniques are crucial for objectives such as improving image quality, reducing noise, increasing contrast, registering images, and extracting features. Figure 7 represents the steps for the post-processing stage.

Median filtering is used to reduce noise in the images. It replaces the value of each pixel with the median value of the neighbouring pixels. This technique is effective for removing salt-and-pepper noise while preserving edges.

Thresholding converts a grayscale image to a binary image. Pixels with intensity above a certain threshold are set to the maximum value (white), and pixels below are set to the minimum value (black). The threshold value defined was 0.5, which determines the cut-off point for binarizing the grayscale image. This means that any pixel value greater than 0.5 will be set to 1 (white), and any pixel value less than or equal to 0.5 will be set to 0 (black).

### 4.5. Experimental Specifications

The experiments aim to simulate the gradual exposure of models to new diverse data, a scenario common in clinical settings, providing advantages such as enhancement in the adaptability of the models, improvement in the training efficiency, and assessment of the generalization capabilities. The methodology for the experiments involves systematically introducing private data to a dataset with two public datasets into the training and validation processes, carefully monitoring changes in model performance across new data insertion. For computational performance considerations, the images were resized to 256×256 pixels. Table 3 shows the model and respective dataset used in each experiment.

### 4.6. Model Configuration and Hyperparameters

For all experiments, the configuration included training the models for a maximum of 100 epochs, with a learning rate set to $1\times 10^{-4}$. The training process was monitored using an early stopping mechanism based on the validation loss to prevent overfitting. Early stopping was triggered if there was no improvement in the validation loss for 5 consecutive epochs. The batch size for training was set to 8, balancing computational efficiency and model performance.

### 4.7. Evaluation

The evaluation metrics used to assess the performance of the models include loss, accuracy, precision, DSC, AOE, ASSD, and MSSD [24]. Each of these metrics provides insights into the performance of the models. However, in a clinical application for liver segmentation in CT scans, the emphasis should be placed on the latter four metrics. DSC evaluates the overall overlap accuracy, while AOE assesses precision in boundary delineation. On the other hand, ASSD and MSSD provide detailed insights into surface accuracy and the worst-case errors, respectively.

For the context of this project, these metrics were prioritized when evaluating model performance against accuracy and precision due to the fact that accuracy is highly influenced by class imbalance and can be misleading in scenarios where the liver constitutes a small part of the CT scan, leading to an overestimation of the performance. Consequently, although these two metrics are important, they must be combined with other performance measures to ensure a thorough evaluation, addressing both average performance and critical edge cases, which is essential for effective and meaningful segmentation of the liver in clinical practice.

## 5. Results

This section presents both the quantitative (Section 5.1) and qualitative (Section 5.2) results obtained by the U-Net, ResUNet++, FCN, and BerDiff models for the different experiments conducted. An analysis of the duration of the training phase for each model is also provided in Section 5.3. While a dataset-wise perspective is presented here, a model-wise one is given in Appendix A.

### 5.1. Quantitative Results

This sub-section compares the results obtained across all the experiments for each dataset (Section 4.3) using the U-Net, ResUNet++, FCN, and BerDiff models. The performance on unseen data is presented as follows: Table 4 covers dataset 1, Table 5 for dataset 2, Table 6 for dataset 3, Table 7 for dataset 4, and Table 8 for dataset 5.

### 5.2. Qualitative Results

**Figure 8:**
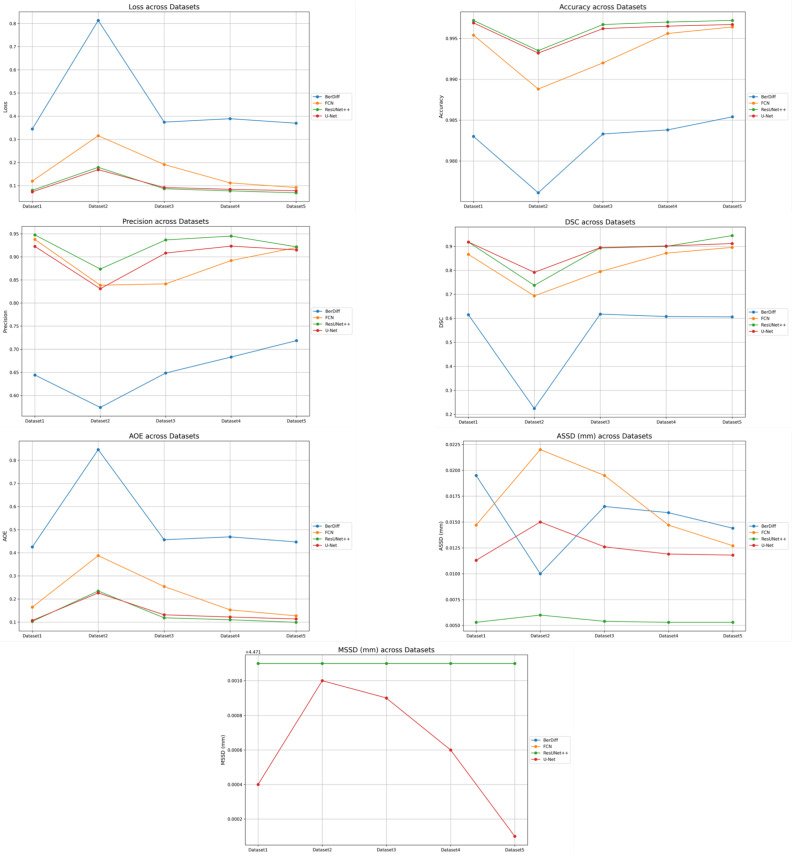
Performance values for each model across all datasets on unseen data: loss, accuracy, precision, DSC, AOE, ASSD, and MSSD.

This sub-section presents a comparison of the results obtained for the experiments for each dataset (Section 4.3) training the U-Net, ResUNet++, FCN, and BerDiff models. A general overview of the values for each metric during the validation of each model across all the datasets on unseen data can be seen in Figure 8.

**Figure 9:**
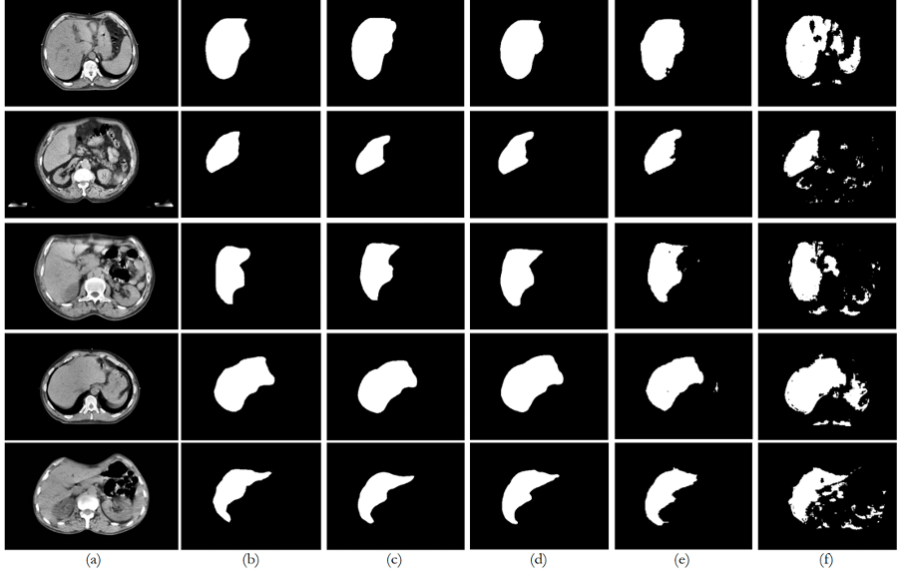
Comparison of the performance from each model for dataset 1 on unseen data: (**a**) original image; (**b**) ground truth; (**c**) U-Net outcome; (**d**) ResUNet++ outcome; (**e**) FCN outcome; (**f**) BerDiff outcome.

**Figure 10:**
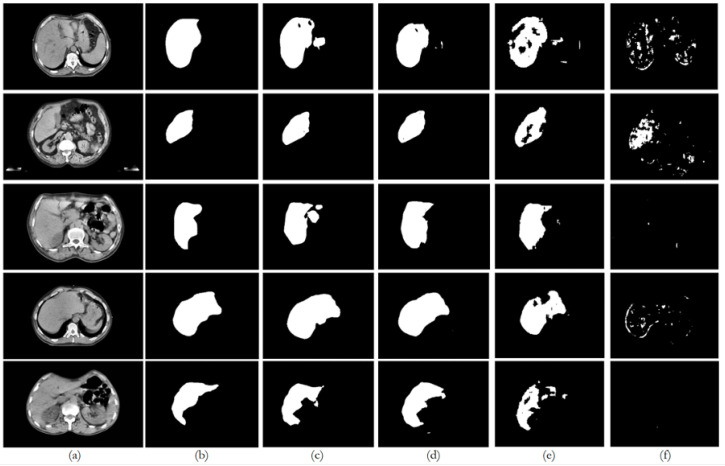
Comparison of the performance from each model for dataset 2 on unseen data: (**a**) original image; (**b**) ground truth; (**c**) U-Net outcome; (**d**) ResUNet++ outcome; (**e**) FCN outcome; (**f**) BerDiff outcome.

**Figure 11:**
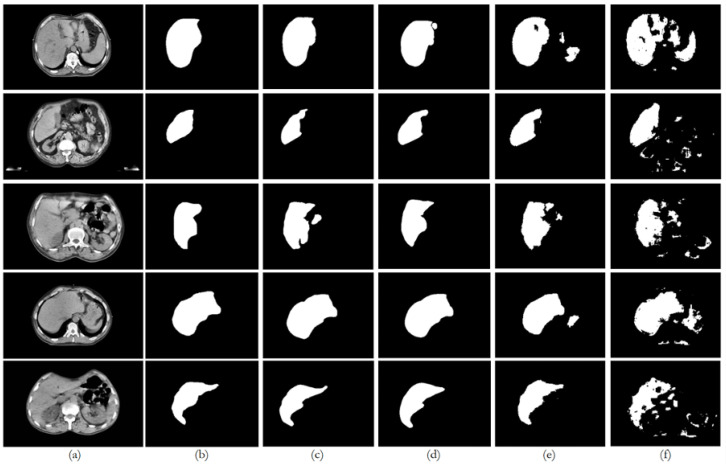
Comparison of the performance from each model for dataset 3 on unseen data: (**a**) original image; (**b**) ground truth; (**c**) U-Net outcome; (**d**) ResUNet++ outcome; (**e**) FCN outcome; (**f**) BerDiff outcome.

**Figure 12:**
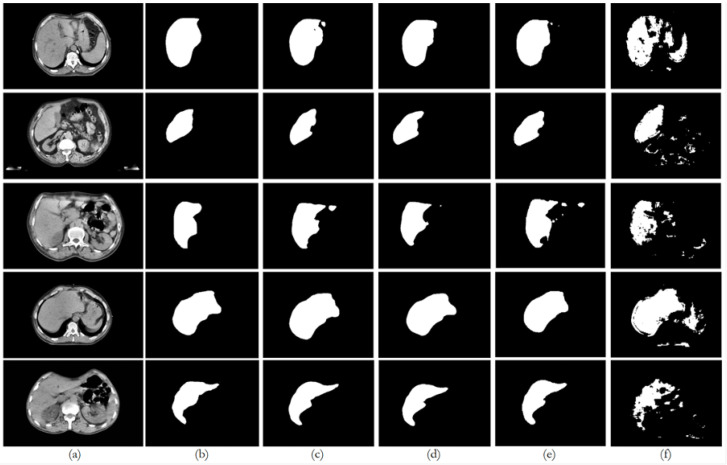
Comparison of the performance from each model for dataset 4 on unseen data: (**a**) original image; (**b**) ground truth; (**c**) U-Net outcome; (**d**) ResUNet++ outcome; (**e**) FCN outcome; (**f**) BerDiff outcome.

**Figure 13:**
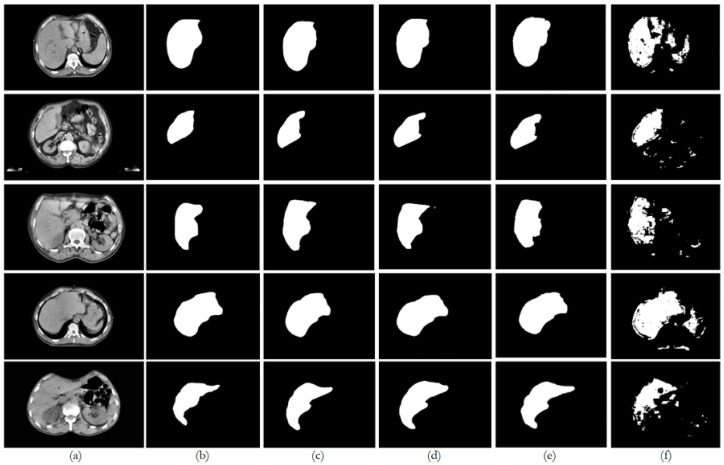
Comparison of the performance from each model for dataset 5 on unseen data: (**a**) original image; (**b**) ground truth; (**c**) U-Net outcome; (**d**) ResUNet++ outcome; (**e**) FCN outcome; (**f**) BerDiff outcome.

Figure 9, Figure 10, Figure 11, Figure 12 and Figure 13 illustrate, respectively, the results obtained in the experiments using datasets 1, 2, 3, 4, and 5, comparing the results from the inferences made on unseen data for the experiments conducted with the five different datasets. Each figure provides an overview of the performance achieved by the models, offering a direct comparison between the different approaches, highlighting the influence of each dataset on the behavior and effectiveness of the tested models, thus demonstrating the robustness and adaptability of the models across different datasets.

### 5.3. Model Training Duration

Table 9 shows the training and validation times for each experiment. ResUNet++ presented the best model performance, achieving the best results despite having the longest per-epoch training time (31–33 min). Its total training times ranged from 3 h:50 m to 9 h:20 m, which is considerable but justified by its superior performance. The ability of the model to converge in fewer epochs (7–17) demonstrates its efficiency in learning from the data. U-Net, the second-best performer, showed consistent speed performance, with each epoch taking only 5–6 min. Its total training times fell between 3 h:00 m and 4 h:05 m, making it the most time-efficient among the models, with the best segmentation studied within the context of this project. The FCN, while presenting lower results than ResUNet++ and U-Net, still offered reasonable results. Its training times varied considerably, from 4 h:37 m to 13 h:18 m, with each epoch lasting 15–17 min. BerDiff, implemented with simplifications to the model, showed the lowest performance among the four. However, it also demonstrated the quickest training times, ranging from 2 h:14 m to 4 h:28 m, with each epoch taking about 10–12 min.

Based on the results presented, the impact of early stopping is visible across all the models, leading to variable total training times. This variability underscores the importance of considering both the potential for early convergence and the per-epoch training time when selecting a model for a given task.

While some models might have shorter per-epoch times or fewer total epochs, this does not necessarily translate to improved performance. The efficiency of a model should be judged not just on its training time but also on its final performance metrics and ability to generalize to unseen data.

## 6. Discussion

This paper presented an approach to compare the capability of different segmentation models to perform liver segmentation, as well as to evaluate the generalization skills of each of the models. This sub-section analyzes the performance across the five different dataset combinations, representing a test of *PDI*, as depicted in the plots in Figure 8 illustrates the trends in quantitative evaluation metrics, and Figure 9, Figure 10, Figure 11, Figure 12 and Figure 13 show the segmentation results in comparison with the ground truth. The model-wise discussion is included in Appendix A.3.

### 6.1. Dataset 1

The private dataset (experiments E1, E6, E11, and E16) on the U-Net and ResUNet++ models showed excellent performance, both of them with accuracy above 0.99 and DSC scores above 0.91 for both training and unseen data, with high DSCs indicating good overlap between the predictions and ground truth, as well as low ASSDs, suggesting precise boundary delineation, as can be seen in the respective qualitative results. It is also worth mentioning that the MSSD value did not show variation for the different models, with a variation after several decimal digits. The FCN performed well but slightly below U-Net and ResUNet++, with a DSC score of 0.8666 on unseen data. BerDiff struggled with this dataset, achieving a DSC score of only 0.6149 on unseen data. It is also visually evident that the performance of the other models differs from that of BerDiff through the qualitative results.

### 6.2. Dataset 2

For the public datasets (experiments E2, E7, E12, and E17), all the models showed a slight decrease in performance, indicating some challenges in generalization. U-Net and ResUNet++ maintained relatively high performance, with DSC scores above 0.73 on unseen data. The FCN’s performance dropped more, with a DSC score of 0.6933 on unseen data. BerDiff showed a pronounced decrease in performance, with a DSC score of only 0.2243 on unseen data, making it notable for its lower performance on metrics such as loss, which was higher than the rest of the models.

### 6.3. Datasets 3, 4, and 5

For the *PDI* mixed datasets (experiments E3–E5, E8–E10, E13–E15, and E18–E20), the U-Net and ResUNet++ models showed improved performance as more data were added, with the best results typically seen in the experiments with datasets 4 and 5. ResUNet++ showed slightly better generalization, with more consistent improvements across *PDI*, presenting the DSC value increasing from 0.8932 to 0.9449 and AOE decreasing from 0.1185 to 0.0990, and maintained the lowest ASSD throughout (0.0053–0.0054 mm), suggesting superior boundary delineation. The FCN model showed improvements with *PDI*, but the gains were less pronounced compared to U-Net and ResUNet++, which is consistent with the behavior of the preceding datasets and the previous experiences. The model’s performance stabilized in the later experiments, suggesting it might benefit from even more data. The BerDiff model also presented some improvements with *PDI*, but its performance remained below the other models. The BerDiff model’s DSC scores on unseen data improved from 0.2243 in E17 to 0.6058 in E20, showing that it benefits from more diverse data.

Despite having initially considered performing data augmentation during the data preparation phase, the process of generalization tests through *PDI* turned out to be a process of increasing the number of input data, the difference being that it provides more benefits than the usual data augmentation process in which the variations may not be as visible as, for example, different images.

### 6.4. Pre-Processing and Post-Processing Impact

All the models underwent the same pre-processing and post-processing stages, allowing for a fair comparison of their intrinsic generalization capabilities. The consistent MSSD values (approximately 4.4721 mm) across all the models and experiments indicate that the post-processing stage effectively standardized the maximum boundary distances, suggesting that, while different CT scan sources affect the overall performance, they do not notably impact the maximum error in boundary delineation. This consistency in MSSD highlights the importance of post-processing in ensuring comparable results across different models and datasets. The pre-processing stage likely contributed to the models’ ability to handle diverse datasets, as evidenced by the improvements seen with *PDI*. However, the varying degrees of performance drop when moving from the private dataset to public datasets indicate that the models have different inherent capabilities in leveraging pre-processed data for generalization.

### 6.5. Private Data Incrementalization

The generalization tests through *PDI* revealed that U-Net and ResUNet++ are the most promising models for the given segmentation tasks. They showed the best performance across different dataset combinations and demonstrated good generalization capabilities. The FCN, while performing well, consistently lagged behind U-Net and ResUNet++, suggesting that it might benefit from architectural improvements or more extensive training. BerDiff’s poor performance across all the experiments indicates that it may not be suitable for these specific segmentation tasks without substantial modifications, which will probably correspond to the architecture of the original model. All the models showed variations in performance when moving from the private dataset to public datasets, indicating that the source and nature of CT scans influence model performance. For example, U-Net’s DSC dropped from 0.9182 on the private dataset to 0.7915 on the public datasets, showing that the characteristics of different CT scan sources can greatly affect segmentation performance.

The models generally performed better on the private dataset compared to public datasets, suggesting that CT scans from different sources (e.g., different machines, protocols, or patient populations) present generalization challenges, highlighting the importance of diverse training data that represent various CT scan sources to improve model robustness.

As more diverse CT scan data were added (combining private and public datasets), all the models showed improvements in performance. An example of this behavior is the ResUNet++ model, whose DSC value increased from 0.7374 on the public datasets alone to 0.9449 when combining the private and public datasets, indicating that a mix of CT scan sources enhances model performance.

The ASSD metric, which is sensitive to boundary precision, showed variations across different dataset combinations. This suggests that different CT scan sources may affect the models’ ability to precisely delineate organ boundaries, possibly due to variations in image quality, contrast, or resolution.

Different models showed varying levels of sensitivity to CT scan sources. ResUNet++ and U-Net demonstrated better adaptability to diverse CT scan sources compared to the FCN and BerDiff. This suggests that model architecture plays a role in how effectively a model can handle variations in CT scan characteristics.

### 6.6. Summary

Based on the results and the analysis presented above, it is possible to answer the following question: *how does the data source (CT scans) impact the performance of the segmentation models?* The CT scan data source impacts segmentation model performance, affecting accuracy, generalization, and boundary precision. Models perform best when trained on diverse CT scan sources that represent the variability expected in real-world applications. This underscores the importance of creating comprehensive and diverse datasets for training robust segmentation models in medical imaging applications.

To mitigate the impact of data source variability and improve the model performance, it is suggested to incorporate diverse CT scan sources in training data, continuing to implement pre-processing techniques to standardize the input data. Another relevant aspect can be considering domain adaptation techniques to better handle CT scans from new unseen sources. Recent advancements in domain adaptation, such as adversarial training, style transfer, and self-supervised adaptation methods [38,39], offer promising solutions for improving model generalization across different CT scan sources. By leveraging these techniques, models can be made more robust, especially in the context of evolving imaging technologies and varying protocols. Last but not least, regularly update and refine models with new CT scan data to maintain performance across evolving imaging technologies and protocols.

## 7. Conclusions

This study addressed a comprehensive evaluation of four different segmentation models (U-Net, ResUNet++, FCN, and BerDiff) across various datasets to assess their generalization capabilities. This final section includes a summary of the strengths of the work (Section 7.1), its limitations (Section 7.2), and some directions for future paths to pursue (Section 7.3).

### 7.1. Strengths

The paper provides a comparison of the performance of different segmentation models, which, conducted across various dataset combinations, offers deep insights into the generalization capabilities of these models. Particularly noteworthy is the assessment of *PDI*, a data-centric approach that evaluated how models perform with the progressive addition of diverse data. This approach provided valuable insights into the importance of dataset diversity in medical imaging tasks. These results not only validate the effectiveness of the chosen models but also contribute to the broader understanding of model behavior in medical image segmentation tasks.

### 7.2. Limitations

Despite the positive results of the study, several limitations were encountered that are important to acknowledge. An important constraint was the limited computational resources, which restricted the ability to test more complex ANN architectures. This was particularly evident in the case of the BerDiff model, for which we employed a simplified version due to memory limitations. As a result, its performance may not fully reflect its potential, and comparisons with other models should be interpreted with caution. This limitation affects the fairness of the evaluation and aligns with the challenges reported in similar studies under resource constraints. Future work will aim to re-evaluate BerDiff using its full version under more favorable computational settings.

The inability to publicly share the private dataset created for this project due to the ethical considerations stipulated by the hospital’s Ethics Committee limits the reproducibility of the exact results and hinders broader validation by the scientific community. Additionally, there were technical barriers in developing automatic export functions and direct integration with the hospital’s server due to strict security measures in the hospital’s IT infrastructure. These challenges represent the complexities of implementing new technologies in sensitive healthcare environments.

### 7.3. Future Work

For the future, there are numerous avenues that could enhance the impact and capabilities of liver segmentation models. One important direction involves the clinical validation of the segmentation results, addressing not only their technical performance but also their practical utility in medical contexts, such as diagnosis, treatment planning, or follow-up monitoring. Collaborations with medical professionals, such as radiologists and oncologists, will be critical to evaluating the clinical relevance, usability, and integration of the tool within actual hospital workflows [40].

Another interesting task is the implementation of an automatic integration not only with the CT scans from the Radiotherapy Department but more broadly of all the scans archived in the hospital’s PACS, which can improve the workflow integration of the application.

Furthermore, there is potential in adapting the algorithm to accurately segment other organs, such as the lungs [41] or the prostate [42], which could greatly expand the HUC’s capabilities across a wider range of anatomical structures.

From a technical perspective, several enhancements could push the boundaries of the performance of the models. Implementing more advanced data augmentation techniques [43,44] could artificially increase dataset diversity, potentially improving model performance, especially for models like FCNs and modified BerDiff that showed more sensitivity to data variability. Exploring transfer learning techniques could also yield benefits, particularly in improving performance on smaller or more specialized datasets. Further optimization of the ResUNet++ and U-Net models, focusing on maintaining their high performance while reducing the computational requirements, could make these models more accessible in resource-constrained environments.

Advanced ML techniques also offer promising directions for future work. Investigating domain adaptation methods could enhance the ability of the models to generalize across different CT scan sources and protocols, a critical factor in real-world medical imaging scenarios. A longitudinal study assessing how the models perform over time as they are exposed to new real-world data could provide insights into strategies for continuous learning and model updating. Incorporating explainable AI techniques [45] could offer valuable insights into the decision-making processes of the models, potentially increasing trust and adoption among medical professionals. Lastly, exploring ensemble methods that combine the strengths of different models could push the boundaries of segmentation accuracy and robustness even further. 

## Figures and Tables

**Table 1:** Comparison between the datasets used.

Dataset	Format	Number of Scans	Number of Slices per Scan	Total Number of Slices	Resolution
Private dataset	DICOM	246	20 to 132	11,175	512×512
3D-IRCADb01 [26]	DICOM	20	74 to 260	2843	512×512
CHAOS [27]	DICOM	40	77 to 105	2915	512×512

**Table 2:** Demographic profile of the patients from the private dataset.

Mean Age (Years)	Standard Deviation (Years)	Minimum Age (Years)	Median Age (Years)	Maximum Age (Years)
71.67	11.91	11	73	99

**Table 3:** Configuration of the performed experiments.

	D1	D2	D3	D4	D5
**U-Net**	E1	E2	E3	E4	E5
**ResUNet++**	E6	E7	E8	E9	E10
**FCN**	E11	E12	E13	E14	E15
**BerDiff**	E16	E17	E18	E19	E20

**Table 4:** Comparison of the performance from each model on unseen data from dataset 1.

Model	Experiment	Loss	Accuracy	Precision	DSC	AOE	ASSD (mm)	MSSD (mm)
U-Net	E1	0.0729	0.9969	0.9228	0.9182	0.1069	0.0113	4.4714
ResUNet++	E6	0.0799	0.9972	0.9477	0.9179	0.1029	0.0053	4.4721
FCN	E11	0.1197	0.9954	0.9379	0.8666	0.1647	0.0147	4.4721
BerDiff	E16	0.3446	0.9830	0.6443	0.6149	0.4250	0.0195	4.4721

**Table 5:** Comparison of the performance from each model on unseen data from dataset 2.

Model	Experiment	Loss	Accuracy	Precision	DSC	AOE	ASSD (mm)	MSSD (mm)
U-Net	E2	0.1689	0.9932	0.8310	0.7915	0.2262	0.0150	4.4720
ResUNet++	E7	0.1790	0.9935	0.8737	0.7374	0.2345	0.0060	4.4721
FCN	E12	0.3155	0.9888	0.8386	0.6933	0.3875	0.0220	4.4721
BerDiff	E17	0.8135	0.9761	0.5739	0.2243	0.8465	0.0100	4.4721

**Table 6:** Comparison of the performance from each model on unseen data from dataset 3.

Model	Experiment	Loss	Accuracy	Precision	DSC	AOE	ASSD (mm)	MSSD (mm)
U-Net	E3	0.0918	0.9962	0.9084	0.8946	0.1315	0.0126	4.4719
ResUNet++	E8	0.0866	0.9967	0.9368	0.8932	0.1185	0.0054	4.4721
FCN	E13	0.1912	0.9920	0.8415	0.7948	0.2540	0.0195	4.4721
BerDiff	E18	0.3743	0.9833	0.6486	0.6174	0.4565	0.0165	4.4721

**Table 7:** Comparison of the performance from each model on unseen data from dataset 4.

Model	Experiment	Loss	Accuracy	Precision	DSC	AOE	ASSD (mm)	MSSD (mm)
U-Net	E4	0.0841	0.9965	0.9234	0.9015	0.1219	0.0119	4.4716
ResUNet++	E9	0.0772	0.9970	0.9452	0.9000	0.1102	0.0053	4.4721
FCN	E14	0.1117	0.9956	0.8921	0.8716	0.1529	0.0147	4.4721
BerDiff	E19	0.3890	0.9838	0.6833	0.6073	0.4689	0.0159	4.4721

**Table 8:** Comparison of the performance from each model on unseen data from dataset 5.

Model	Experiment	Loss	Accuracy	Precision	DSC	AOE	ASSD (mm)	MSSD (mm)
U-Net	E5	0.0780	0.9967	0.9151	0.9118	0.1135	0.0118	4.4711
ResUNet++	E10	0.0691	0.9972	0.9218	0.9449	0.0990	0.0053	4.4721
FCN	E15	0.0920	0.9964	0.9204	0.8956	0.1271	0.0127	4.4721
BerDiff	E20	0.3696	0.9854	0.7184	0.6058	0.4469	0.0144	4.4721

**Table 9:** Training and validation duration for each experiment.

Experiment	Model	Number of Epochs	Training and Validation Time
E1	U-Net	36	3 h:30 m
E2		42	4 h:05 m
E3		31	3 h:00 m
E4		36	3 h:30 m
E5		40	3 h:53 m
E6	ResUNet++	10	5 h:29 m
E7		16	8 h:47 m
E8		10	5 h:29 m
E9		17	9 h:20 m
E10		7	3 h:50 m
E11	FCN	37	10 h:03 m
E12		49	13 h:18 m
E13		28	7 h:36 m
E14		22	5 h:58 m
E15		17	4 h:37 m
E16	BerDiff	17	3 h:09 m
E17		21	3 h:54 m
E18		24	4 h:28 m
E19		14	2 h:36 m
E20		12	2 h:14 m

**Figure A1:**
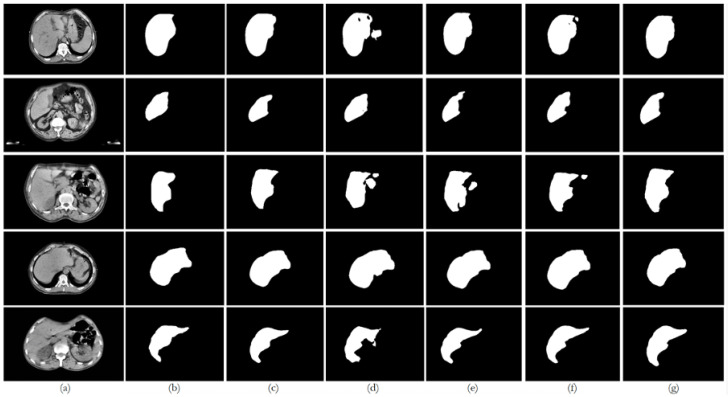
Performance of the U-Net experiments on unseen data: (**a**) original image; (**b**) ground truth; (**c**) segmentation outcome with E1; (**d**) segmentation outcome with E2; (**e**) segmentation outcome with E3; (**f**) segmentation outcome with E4; (**g**) segmentation outcome with E5.

**Figure A2:**
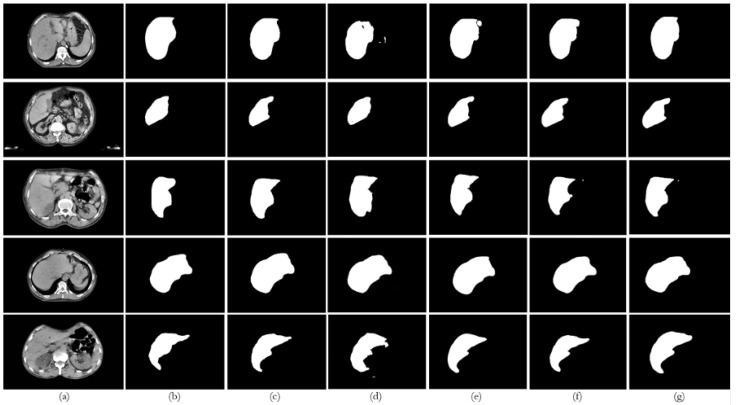
Performance of the ResUNet++ experiments on unseen data: (**a**) original image; (**b**) ground truth; (**c**) segmentation outcome with E6; (**d**) segmentation outcome with E7; (**e**) segmentation outcome with E8; (**f**) segmentation outcome with E9; (**g**) segmentation outcome with E10.

**Figure A3:**
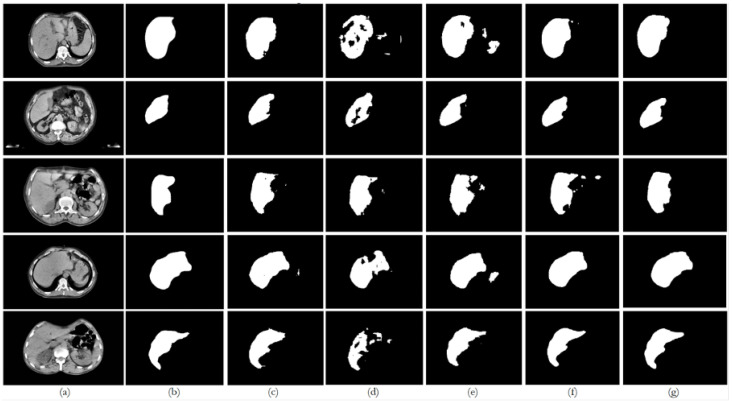
Performance of the FCN experiments on unseen data: (**a**) original image; (**b**) ground truth; (**c**) segmentation outcome with E11; (**d**) segmentation outcome with E12; (**e**) segmentation outcome with E13; (**f**) segmentation outcome with E14; (**g**) segmentation outcome with E15.

**Figure A4:**
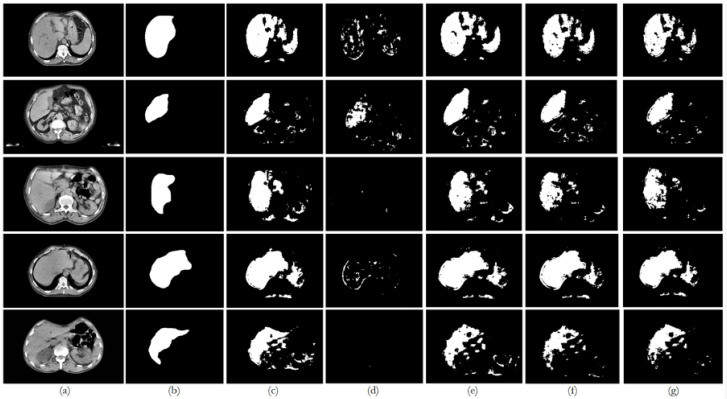
Performance of the BerDiff experiments on unseen data: (**a**) original image; (**b**) ground truth; (**c**) segmentation outcome with E16; (**d**) segmentation outcome with E17; (**e**) segmentation outcome with E18; (**f**) segmentation outcome with E19; (**g**) segmentation outcome with E20.

## Data Availability

The original contributions presented in the study are included in the article, further inquiries can be directed to the corresponding authors.

## Appendix A. Model-Wise Perspective of the Results

This section presents both quantitative (Appendix A.1) and qualitative (Appendix A.2) results in a model-wise perspective and the respective Discussion (Appendix A.3).

### Appendix A.1. Quantitative Results

The evaluation results for *PDI* across different models on unseen data are summarized as follows: experiments using the U-Net model (E1–E5) are detailed in Table A1, those using the ResUNet++ model (E6–E10) in Table A2, results for the FCN model (E11–E15) are presented in Table A3, and experiments with the BerDiff model (E16–E20) are shown in Table A4.

**Table A1:** Performance of the U-Net experiments on unseen data.

Experiment	Loss	Accuracy	Precision	DSC	AOE	ASSD (mm)	MSSD (mm)
E1	0.0729	0.9969	0.9228	0.9182	0.1069	0.0113	4.4714
E2	0.1689	0.9932	0.8310	0.7915	0.2262	0.0150	4.4720
E3	0.0918	0.9962	0.9084	0.8946	0.1315	0.0126	4.4719
E4	0.0841	0.9965	0.9234	0.9015	0.1219	0.0119	4.4716
E5	0.0780	0.9967	0.9151	0.9118	0.1135	0.0118	4.4711

**Table A2:** Performance of the ResUNet++ experiments on unseen data.

Experiment	Loss	Accuracy	Precision	DSC	AOE	ASSD (mm)	MSSD (mm)
E6	0.0799	0.9972	0.9477	0.9179	0.1029	0.0053	4.4721
E7	0.1790	0.9935	0.8737	0.7374	0.2345	0.0060	4.4721
E8	0.0866	0.9967	0.9368	0.8932	0.1185	0.0054	4.4721
E9	0.0772	0.9970	0.9452	0.9000	0.1102	0.0053	4.4721
E10	0.0691	0.9972	0.9218	0.9449	0.0990	0.0053	4.4721

**Table A3:** Performance of the FCN experiments on unseen data.

Experiment	Loss	Accuracy	Precision	DSC	AOE	ASSD (mm)	MSSD (mm)
E11	0.1197	0.9954	0.9379	0.8666	0.1647	0.0147	4.4721
E12	0.3155	0.9888	0.8386	0.6933	0.3875	0.0220	4.4721
E13	0.1912	0.9920	0.8415	0.7948	0.2540	0.0195	4.4721
E14	0.1117	0.9956	0.8921	0.8716	0.1529	0.0147	4.4721
E15	0.0920	0.9964	0.9204	0.8956	0.1271	0.0127	4.4721

**Table A4:** Performance of the BerDiff experiments on unseen data.

Experiment	Loss	Accuracy	Precision	DSC	AOE	ASSD (mm)	MSSD (mm)
E16	0.3446	0.9830	0.6443	0.6149	0.4250	0.0195	4.4721
E17	0.8135	0.9761	0.5739	0.2243	0.8465	0.0100	4.4721
E18	0.3743	0.9833	0.6486	0.6174	0.4565	0.0165	4.4721
E19	0.3890	0.9838	0.6833	0.6073	0.4689	0.0159	4.4721
E20	0.3696	0.9854	0.7184	0.6058	0.4469	0.0144	4.4721

### Appendix A.2. Qualitative Results

This sub-section presents a comparative analysis of the results obtained in the training experiments for the U-Net, ResUNet++, FCN, and BerDiff models, exploring the impact of *PDI*. Figure A1, Figure A2, Figure A3 and Figure A4 illustrate, respectively, the inferences on unseen data for the series of experiments E1–E5 (U-Net), E6–E10 (ResUNet++), E11–E15 (FCN), and E16–E20 (BerDiff), providing a comprehensive view of the performance of each model with datasets from different sources and varying dataset size.

### Appendix A.3. Discussion

This sub-section presents an analysis of the results presented by the various models, focusing on the ability of the models to adapt and generalize.

#### Appendix A.3.1. U-Net

The U-Net model presented versatility and robustness across all the datasets, as illustrated in Figure A1. This figure shows the segmentation outcomes for U-Net across experiments. Its consistently high DSC scores, even when applied to public datasets (maintaining DSC > 0.79), underscore its strong generalization capacity. This model exhibited a good ability to adapt and improve with the *PDI*, as evidenced by steady enhancements in both DSC and AOE metrics. Perhaps most notably, U-Net’s boundary delineation capabilities proved exceptionally precise and stable, maintaining a remarkable ASSD ranging from 0.0113 to 0.0150 mm across all experiments. This consistent performance in boundary precision, as well as its strong overall segmentation accuracy and adaptability, make U-Net a highly reliable and efficient choice for the objectives of the project.

#### Appendix A.3.2. ResUNet++

The ResUNet++ model showed the best performance in the comparative study, showcasing superior capabilities across all key performance indicators (as shown in Figure A2). This model demonstrated the most robust generalization capacity among all the models evaluated. A relevant aspect of ResUNet++’s performance was its unparalleled precision in boundary delineation, consistently maintaining the lowest ASSD throughout all experiments. This exceptional boundary accuracy, combined with its ability to achieve the highest DSC of 0.9449 and the lowest AOE of 0.0990 in the final experiment (E10), underscore its superior segmentation quality and precise liver localization capabilities.

#### Appendix A.3.3. FCN

The FCN model demonstrated moderate generalization capacity, showing strong capabilities when applied to the private dataset, but presented more pronounced performance degradation when transitioning to public datasets, especially in comparison to the more robust U-Net and ResUNet++ models, as illustrated in Figure A3. This suggests a higher sensitivity to variations in data sources and characteristics. However, FCN showed a commendable capacity for improvement with the *PDI*, indicating its potential for enhanced performance given sufficiently varied input. A consistent observation across experiments was FCN’s higher ASSD compared to U-Net and ResUNet++, pointing to less precise boundary delineation capabilities. This suggests that, while FCNs can produce good overall segmentations, they may struggle with the fine details of organ boundaries.

#### Appendix A.3.4. BerDiff

The BerDiff model consistently underperformed relative to the other models, presenting challenges across all experiments, as shown in Figure A4. Its performance was particularly problematic when generalizing to public datasets, evidenced by a decline in DSC from 0.6149 to 0.2243. This substantial drop highlights the modified BerDiff’s limited ability to adapt to varied data sources and characteristics. Interestingly, despite its overall poor performance, BerDiff demonstrated the highest relative improvement with *PDI*. This observation suggests that the model may require substantially more extensive and diverse training data to achieve competitive performance levels. However, even with these improvements, BerDiff’s performance remained below that of the other models in the study.

Although the only evidence of the original BerDiff model’s performance can be found in the authors’ original paper [37], it is possible to verify that, possibly due to the simplification of the model described in Section 4.4.2, it performed poorer than the other models. In any case, for contexts such as this project (without sufficient conditions and computational resources to train the model with the original architecture), the use of such complex and heavy models is not advantageous since there are models that seem to have a simpler architecture but remain very powerful for their intended purpose.

#### Appendix A.3.5. Models’ Generalization Capacity

ResUNet++ demonstrated the best generalization capacity, showing the most consistent performance across different datasets and the most improvements *PDI*. U-Net also showed strong generalization capabilities, maintaining high DSC scores across experiments and showing steady improvements with more data. FCN presented moderate generalization capacity, with more pronounced performance drops on public datasets but good improvements with *PDI*. However, the simplified BerDiff model demonstrated poor generalization capacity, struggling with public datasets and showing limited improvement even with additional data.

#### Appendix A.3.6. Summary

Based on the results from all the experiments, it is possible to answer the question: *which is the most efficient automatic segmentation model for the case study within the chosen algorithms?* The most efficient automatic segmentation models for this case study are U-Net and ResUNet++. However, ResUNet++ slightly edges out U-Net as the most efficient model overall. While both models performed exceptionally well, ResUNet++ showed slightly better generalization and more consistent improvements across *PDI*. Also, ResUNet++ presented a balance between segmentation performance and relatively efficient training time despite longer per-epoch duration. Its superior performance in boundary precision (as indicated by the lowest ASSD) and overall segmentation quality (highest DSC in the final experiment) make it the most efficient model for this case study.

The choice between ResUNet++ and U-Net might depend on specific use case requirements. If computational resources are a constraint, U-Net might be preferable as it is generally less complex than ResUNet++. However, if the highest possible accuracy and generalization are the primary concerns, ResUNet++ would be the top choice.
